# Biomarkers for pneumonia after major trauma: A systematic review and meta-analysis

**DOI:** 10.1177/17511437251344068

**Published:** 2025-06-13

**Authors:** Fiona Howroyd, Amanda Veiga Sardeli, Fang Gao Smith, Tonny Veenith, Niharika A Duggal, Zubair Ahmed

**Affiliations:** 1 Department of Inflammation and Ageing, School of Infection, Inflammation and Immunology, College of Medicine and Health, University of Birmingham, Birmingham, UK; 2 Critical Care Department, University Hospitals Birmingham NHS Foundation Trust, Birmingham, UK; 3 Critical Care Department, Royal Wolverhampton Hospital, Wolverhampton, UK; 4 Institute of Acute Care, University of Wolverhampton and Royal Wolverhampton Hospital, Wolverhampton, UK

**Keywords:** Major injury, trauma, pneumonia, blood biomarkers, respiratory infection

## Abstract

**Background:**

Major trauma is a significant global health issue. Pneumonia poses an additional risk for morbidity and mortality after major trauma yet identifying pneumonia remains challenging in clinical practice. This systematic review aims to evaluate blood-based biomarkers for pneumonia in major trauma patients.

**Methods:**

The search was performed across four databases up to November 18th 2024, including primary studies investigating blood-based biomarkers associated with pneumonia in adults hospitalised after major trauma (PROSPERO CRD42024542059). Risk of bias was assessed using the ROBINS-E tool and meta-analysis was performed of pooled data.

**Results:**

Among 20 included studies, with a total of 4316 participants, the pooled mean pneumonia rate was 32.7% (23.5%–43.4%). Seventy biomarkers for post-operative pneumonia were identified, with meta-analysis possible for 12 of the reported biomarkers. At admission interleukin (IL)-6 (standardised mean difference: 1.41 (0.04–2.77), *p* = 0.04), cytokeratin fragment 21-1 (CYFRA21-1; 0.53 (0.19–0.86), *p* = 0.002) and leucocyte count (0.28 (0.05–0.50), *p* = 0.01) were higher in patients who developed pneumonia. During hospitalisation, patients with pneumonia had significantly higher IL-10 (4.42 (3.89–4.95), *p* > 0.001) and neutrophil oxidative burst capacity (1.52 (0.96–2.09), *p* > 0.001) at day 1, CYFRA21-1 at day 2 (0.43 (0.10–0.76), *p* = 0.01), IL-6 at day 3 (3.11 (2.66–3.55), *p* > 0.001) and day 5 (0.57 (0.05–1.09), *p* = 0.03) and CRP at day 4 (1.87 (1.51–2.24), *p* > 0.001), day 5 (1.38 (1.03–1.72), *p* > 0.001), day 6 (0.74 (0.42–1.06), *p* > 0.001) and day 7 (0.87 (0.12–1.63), *p* = 0.02). Across the included studies, 85% exhibited some concerns to very high risk of bias.

**Conclusions:**

While we identified potential candidate biomarkers for pneumonia in major trauma patients, the high heterogeneity across trauma populations, clinical diagnostic tools and biomarker testing methods warrants further high-quality studies to confirm their clinical value.

## Graphical abstract


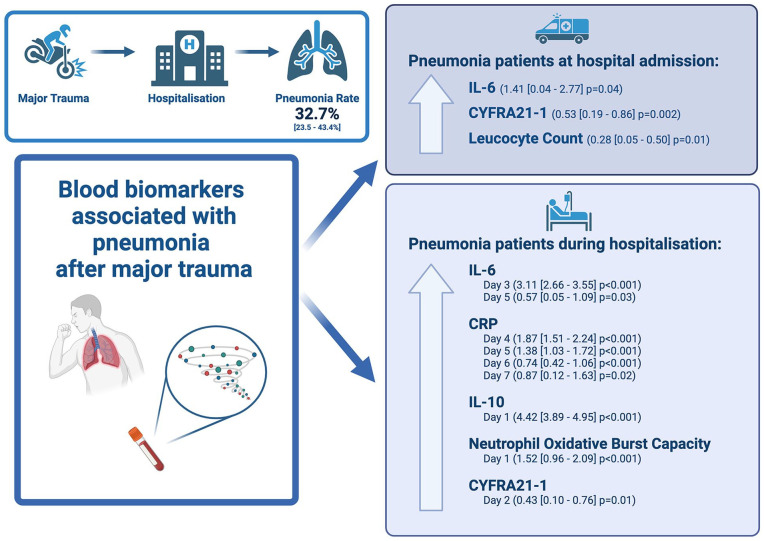


## Introduction

Major trauma is defined as a significant injury, or group of injuries, that have the potential to be life-changing or life-threatening and is a leading cause of death and disability worldwide in all age groups.^1,2^ In adults aged under 50 years, road injuries are the leading global cause of disability-adjusted-life years, with self-harm and interpersonal violence also ranking in the top 15 mechanisms.^3^ Although previously viewed as a disease burden predominantly affecting young males, there is increasing prevalence of major trauma within elderly populations, primarily associated with falls and low impact trauma.^3,4^ Each year there are at least 20,000 cases of major trauma in England, costing the UK National Health Service (NHS) £0.4 billion annually in the acute care setting alone.^5,6^ Affecting a working-age population, major trauma is estimated to have a profound impact on society, with an annual loss of economic output of up to £3.7 billion pounds each year in the UK.^6^

Pneumonia is a broad term encompassing heterogeneous respiratory infections of varying epidemiology, pathology and clinical presentation, commonly classified as hospital-acquired or community-acquired.^7^ Hospital acquired pneumonia (HAP) is an acute, infection of the pulmonary parenchyma that manifests after 48 h of hospital admission.^7^ Nosocomial infections and multiple organ failure, including that attributed to HAP, are the most common secondary causes of death that occur in the days to weeks after primary injury.^8,9^ Reported pneumonia rates after major trauma vary substantially due to heterogenous injury severity and inconsistent diagnostic criteria, yet published statistics suggest at least one quarter of patients are affected.^10–16^ Patients who are particularly susceptible are those with low scores on the Glasgow Coma Scale (GCS) and high Injury Severity Scores (ISS).^8,17^ Such patients commonly require endotracheal intubation and mechanical ventilation and are subsequently at risk of ventilator acquired pneumonia (VAP).^17^ VAP is a subtype of HAP that is defined as pneumonia that occurs at least 48 h after intubation, usually caused by highly resistant pathogens that need treatment with extended-spectrum antibiotics.^7^ The incidence of VAP in trauma patients is four-times greater than in non-trauma patients.^17,18^ Those who develop pneumonia after traumatic injury have increased risk of mortality, longer intensive care unit (ICU) and hospital length of stays and are more likely to be discharged to an ongoing care or rehabilitation facility.^8,17^

The identification of pneumonia risk and onset is challenging. Despite published international guidelines, there is also recognised variability in the diagnosis of pneumonia, which lacks sensitivity and specificity.^7,19^ Traditionally, pneumonia diagnosis relies on a combination of clinical, radiographic and laboratory criteria.^7,8,19,20^ However, many of the common diagnostic tools present challenges in the major trauma population and subsequently increase the risk of over-estimated incidence rates.^7,16^ With the increasing prevalence of nosocomial infections and antimicrobial resistance presenting a global public health threat, it is imperative that diagnostic methods for pneumonia are improved.^21^

Biomarkers provide an objective and quantifiable characteristic of a biological process and include fluid-based biomarkers, such as circulating proteins in serum.^22^ Biomarkers may therefore act as a potential diagnostic method for numerous infections, including pneumonia.^23–25^ Previous meta-analyses have identified potential biomarkers for the diagnosis and prognosis of community acquired pneumonia, yet there are no previous known reports specific to hospital acquired pneumonia or major trauma.^26–29^ Early, standardised and quantifiable methods for identifying pneumonia would reduce antibiotic prescription pressures and subsequently reduce mortality, morbidity and the health economics associated with trauma induced pneumonia. Biomarkers offer an objective solution to improve clinical diagnosis, yet currently, the most effective biomarker for pneumonia after major trauma is unknown. Thus, this systematic review aimed to identify blood-based biomarkers associated with pneumonia after major trauma.

## Materials and methods

The protocol for this systematic review and meta-analysis was registered with the international prospective register of systematic reviewers (PROSPERO; CRD42024542059).^30^ Since protocol publication, there have been a number of changes: (1) the study does not focus upon diagnosis of pneumonia due to extensive heterogeneity in clinical diagnosis methods and biomarker assessment methods, and thus reports biomarkers associated with pneumonia in the title and aims; (2) use of the PECO syntax as the results of the literature search subsequently identified observational, non-interventional studies; (3) sub-group analysis was based upon the time-point of biomarker assessment, at either hospital admission or during hospitalisation; and (4) analysis was conducted using Biostat’s Comprehensive Meta-Analysis software (version 3.0; New Jersey, USA). As this systematic review aimed to identify and evaluate biomarkers associated with pneumonia, rather than for diagnosis, the review has been reported following Preferred Reporting Items for Systematic Reviews and Meta-Analyses (PRISMA) guidelines.^31^

### Search strategy

Four electronic databases PubMed, Cochrane Library, Ovid Medline and EMBASE were used to perform the systematic review and were searched up to 18th November 2024 for published studies from all years. Searches were conducted using a combination of various keywords, synonyms, heading (MeSH) and entry terms for common search strings including ‘pneumonia’ AND ‘biomarkers’ AND ‘major trauma’ within the title and abstract (Supplemental File 1). The ‘PECO’ (Population, Exposure, Comparator and Outcome) syntax framework for non-interventional studies was utilised to formulate and refine the search strategy and aims including; adults hospitalised after major trauma (population), exposed to pneumonia (exposure) compared to those without pneumonia (comparator), investigating blood-based biomarkers (outcome).^32^

### Study screening and selection

Duplicate records amongst the four databases were removed prior to screening. Initial screening of the title and abstracts were completed by two independent reviewers (FH and ZA) using Rayyan.^33^ Shortlisted studies were then reviewed in full text and assessed for eligibility. Any disagreements were resolved by discussion. Only studies identifying blood-based biomarkers for pneumonia after major trauma were considered for this systematic review. There was no limit to the time or location of published studies. Only full-text articles published in the English language and in a peer-reviewed journal were considered, with no limit to the type of clinical study conducted. However, case reports and studies that did not include a comparator control group of patients without pneumonia were excluded.^34^ The eligibility criteria included: (1) human studies; (2) adults (aged 18 years or above); (3) hospitalised after any acute traumatic injury; (4) investigating any blood-based biomarker; (5) reporting mean, standard deviation (SD) and sample size for each biomarker in both pneumonia and no-pneumonia groups; and (6) diagnosing pneumonia during acute hospitalisation after major trauma, using any criteria.

### Data extraction

Data extraction was completed by one reviewer (FH) then checked by a second reviewer (ZA), conflicts were resolved by both reviewers cross-checking against the original text to reach consensus. Data collection of the key characteristics of each included study was performed using Microsoft Excel and included authors, year of publication, location of where the study was conducted, the type of injury sustained, the biomarker assessed and the method for pneumonia diagnosis. Injuries were grouped into sub-types, including polytrauma, neurotrauma or burns. Pneumonia was recorded as VAP or HAP; studies that mentioned patients being ventilated or specified VAP were recorded as VAP and all other studies were classified as HAP. The mean and standard deviation (SD) for each biomarker and sample size for pneumonia and non-pneumonia groups were recorded.

### Risk of bias assessment

A risk of bias assessment was conducted using the ROBINS-E tool to assess the risk of bias in observational studies, estimating the effect of an exposure (i.e. pneumonia) on an outcome (i.e. blood-based biomarkers).^35^ Risk of bias against the seven criteria, as well as overall risk of bias were judged by two authors independently (FH and ZA). The judgement of the overall risk of bias was categorised from low risk to very high risk using the ROBINS-E tool algorithm, with a general rule that the overall risk for each study was determined by the domain with the greatest risk of bias judgement. Any discrepancies were resolved through discussion.

### Statistical analysis

Meta-analysis of pooled data was conducted using Biostat’s Comprehensive Meta-Analysis software (version 3.0; New Jersey, USA), employing the continuous data function and random effects model due to high heterogeneity (i.e. *I*^2^ > 50%). The standardised mean difference (SMD), 95% confidence intervals (CI) and *p* values for the test of overall effect, along with *Q* and *I*^2^ statistics were reported for each biomarker when reported in two or more studies. For biomarkers reported in one study, the SMD and CI were recorded, since meta-analysis was not feasible. Analysis was grouped by the time of biomarker assessment, including hospital admission and during hospitalisation, with hospitalisation further sub-grouped according to reported time-points. The Egger test was performed to test for publication bias, in biomarkers that were reported in two or more studies. A complementary analysis of the mean values of commonly reported biomarkers was reported to provide an exploratory observation of clinically meaningful biomarker metrics using GraphPad PRISM^®^ software, with biomarker data converted into standardised units of measure. The pooled rates of pneumonia were analysed using Biostat’s Comprehensive Meta-Analysis software (version 3.0; New Jersey, USA), employing the continuous data function and random effects model. A further complementary analysis of the pneumonia rates reported in each study was performed using GraphPad PRISM^®^ software; comparing mean rates of VAP and HAP by unpaired students *T*-test (parametric test for normally distributed data) and comparing mean pneumonia rates by injury subtype by one-way analysis of variance (ANOVA) with Bonferroni correction. A *p* value of <0.05 was deemed statistically significant.

## Results

**Figure 1.**
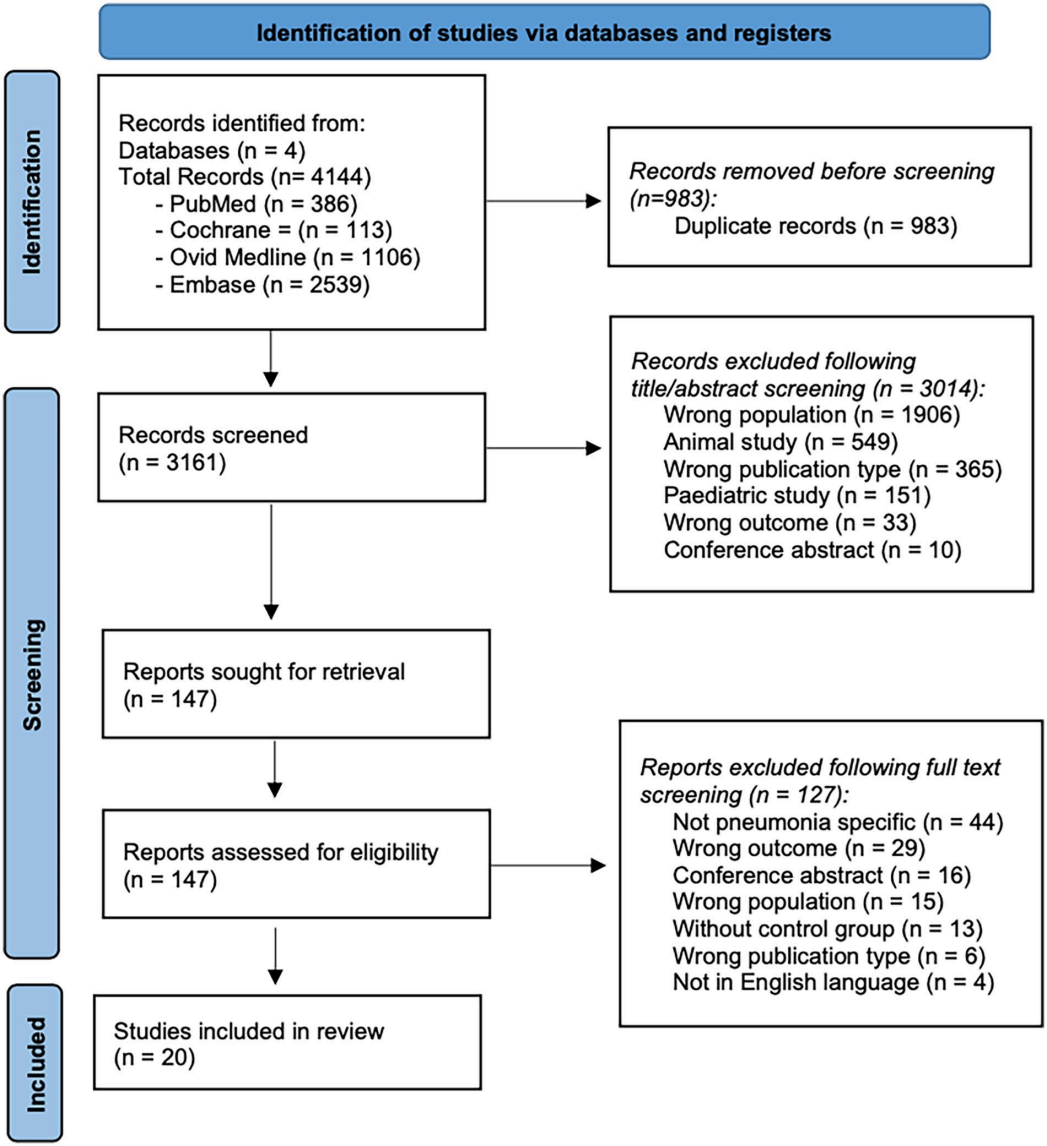
PRISMA flow chart of study selection.

The literature search process including identification, screening and inclusion is presented in Figure 1. The search strategy identified 4144 articles across the four electronic databases. After removing 983 duplicates, 3161 records remained for initial screening. After title and abstract screening, 147 studies remained; with more than half of the studies (*n* = 1906) excluded due to the wrong study population and over 18% (*n* = 549) were animal studies. After full text reading a further 127 studies were excluded, with the wrong study outcome being the main reason for exclusion in over a third of the studies (*n* = 44). Thus, 20 studies were included in the final review. Inter-rater reliability was 96%.

A summary of the key characteristics of each study are detailed in Table 1, including demographics, injury sub-type, pneumonia rates and tested biomarkers. There was a total of 4316 hospitalised patients with major trauma across 20 studies. The majority (92.6%) of all patients sustained polytrauma (*n* = 3998), 4.7% sustained neurotrauma (isolated traumatic brain injury; *n* = 202) and 2.7% sustained burn injuries (*n* = 116).

**Table 1.** Characteristics of the included studies.

Study	Location	Injury type	Patients (*n*)	Pneumonia (*n*)	HAP/VAP	Timepoint of biomarker assessment	Biomarkers in association with pneumonia included in meta-analysis
Baker et al.^40^	USA	Polytrauma	98	72	VAP	During hospitalisation: day 7	C-reactive protein (CRP)
Bouras et al.^41^	France	Neurotrauma	179	89	VAP	At admission: within 24 h after injury	Partial pressure of carbon dioxide (PaCO_2_), leucocyte count, neutrophil count, lymphocyte count, CRP, cortisol total concentration, cortisol free concentration, transcortin, cortisol free/CRP ratio, cortisol total/CRP ratio
Bradley et al.^42^	USA	Polytrauma	73	9	HAP	During hospitalisation: prior to surgery and pneumonia diagnosis	Fibroblast growth factor-basic (FGF-basic), interlukin-2 receptor (IL-2R), interleukin (IL)−6
Cohen et al.^43^	USA	Polytrauma	59	17	VAP	At admission: on arrival to the emergency department. During hospitalisation: at 6 h, 12 h and at day 1	Plasma protein C (PPC), soluble endothelial plasma protein C receptor (SEPCR), soluble thrombomodulin
Fachet et al.^44^	Germany	Polytrauma	317	39	HAP	At admission: on arrival to the emergency department During hospitalisation: day 1–3	IL-6, IL-10
Habib et al.^36^	Egypt	Polytrauma	49	36	VAP	At admission: on intubation. During hospitalisation: at VAP diagnosis and day 3	Neutrophil cluster of differentiating 64 (nCD64), procalcitonin (PCT), CRP
Kowal-Vern et al.^45^	USA	Burns	24	9	HAP	During hospitalisation: at day 1–2 and day 3–6	Antithrombin (AT)
Lumsdaine et al.^46^	Australia	Polytrauma	100	7	HAP	During hospitalisation: pre-operatively and day 1 post	Neutrophil oxidative burst capacity (NOBC)
Menni et al.^47^	Greece	Polytrauma	112	22	VAP	At admission During hospitalisation: day 7	Neutrophil count, lymphocyte count and neutrophil-lymphocyte ratio (NLR)
Mukherjee et al.^48^	USA	Polytrauma	2656	329	VAP	During hospitalisation: 8, 6 and 2 days pre-VAP and 3, 6 and 8–10 days post-VAP	Serum blood glucose concentration
Negrin et al.^49^	Austria	Polytrauma	28	12	HAP	At admission During hospitalisation: day 1, 3, 5, 7, 10, 14, 21	Plasminogen activator inhibitor-1 (PAI-1)
Negrin et al.^37^	Austria	Polytrauma	101	30	HAP	At admission During hospitalisation: day 2	Soluble receptor for advanced glycation-end products (sRAGE), club cell protein 16 (CC16), surfactant protein D (SP-D), cytokeratin fragment 21-1 (CYFRA21-1), Krebs von den Lungen 6/Mucin 1 (KL-6/MUC1)
Ruiz-Castilla et al.^50^	Spain	Burns	24	8	HAP	At admission During hospitalisation: day1	Creatinine, platelet count, bilirubin, CRP, albumin, lactate, receptor for advanced glycation-end products (RAGE), SP-D, IL-6, IL-8, IL-33, Soluble suppression of tumorigenicity-2 (sST2)
Schaefer et al.^38^	Germany	Polytrauma	14	7	HAP	During hospitalisation: day1-2	Lactate, glucose, sodium, bilirubin, creatinine, haemoglobin, white blood cell count (WBC), platelets, CRP. Slope (change in); t-cell immunoglobulin and mucin-domain containing-3 (TIM-3), cluster of differentiating (CD) 27, CD40, herpes virus entry mediator (HVEM), B and T lymphocyte attenuator (BTLA), cytotoxic T lymphocyte antigen-4 (CTLA-4), programmed cell death protein 1 (PD-1), glucocorticoid-induced tumour necrosis factor receptor-related protein (GITR), GITR ligand (GITRL), CD80, CD28, CD86, programmed cell death ligand-1 (PD-L1), toll-like receptor 2 (TLR-2), inducible co-stimulatory molecule (ICOS), lymphocyte activation-gene-3 (LAG-3)
Schlosser et al.^51^	Germany	Neurotrauma	32	15	HAP	At admission: between 2 and 24 h	IL-6
Textoris et al.^52^	France	Polytrauma	165	41	VAP	At admission During hospitalisation: at VAP onset or between day 5 and 10	Creatinine, leucocyte count, haemoglobin, platelet count, fibrinogen, plasma lactate. Gene expression: ALAS2, AHSP, PCSK1, PPBP, SPARC, TNF, IL1B, IL1RN, TREM, IL6R
Trabelsi et al.^53^	Tunisia	Polytrauma	167	67	HAP	During hospitalisation: days 3–9	CRP
Turan et al.^54^	Switzerland	Burns	90	37	HAP	At admission	Selenium-binding protein 1 (SELENBP1)
Vollrath et al.^55^	Germany	Polytrauma	73	20	HAP	At admission: trauma room, ICU arrival, day 0 During hospitalisation: day 2 and day 5	Haemoglobin, CYFRA 21-1, Angiopoetin-2, pentraxin 3, sRAGE, IL-6, IL-10
Wutzler et al.^39^	Germany	Polytrauma	58	20	HAP	During hospitalisation: at pneumonia onset	CC16

HAP: hospital acquired pneumonia; VAP: ventilator acquired pneumonia.

The pooled mean pneumonia rate across the 20 studies was 32.7% (23.5–43.4%). Only four studies reported microbiology results to identify the type of infection attributable to pneumonia, with gram-negative bacteria the most commonly recorded.^36–39^

Eighteen of the studies reported patient age (*n* = 1623), with a mean age of 42 ± 7.9 years (range 22–54 years). Eleven studies reported age in those with and without pneumonia, with no significant differences in age between groups (45 ± 7.9 vs 41 ± 6.7 years, *p* = 0.23). In the 14 studies that reported gender (*n* = 1208), 64% were male (*n* = 774).

### Risk of bias

**Figure 2.**
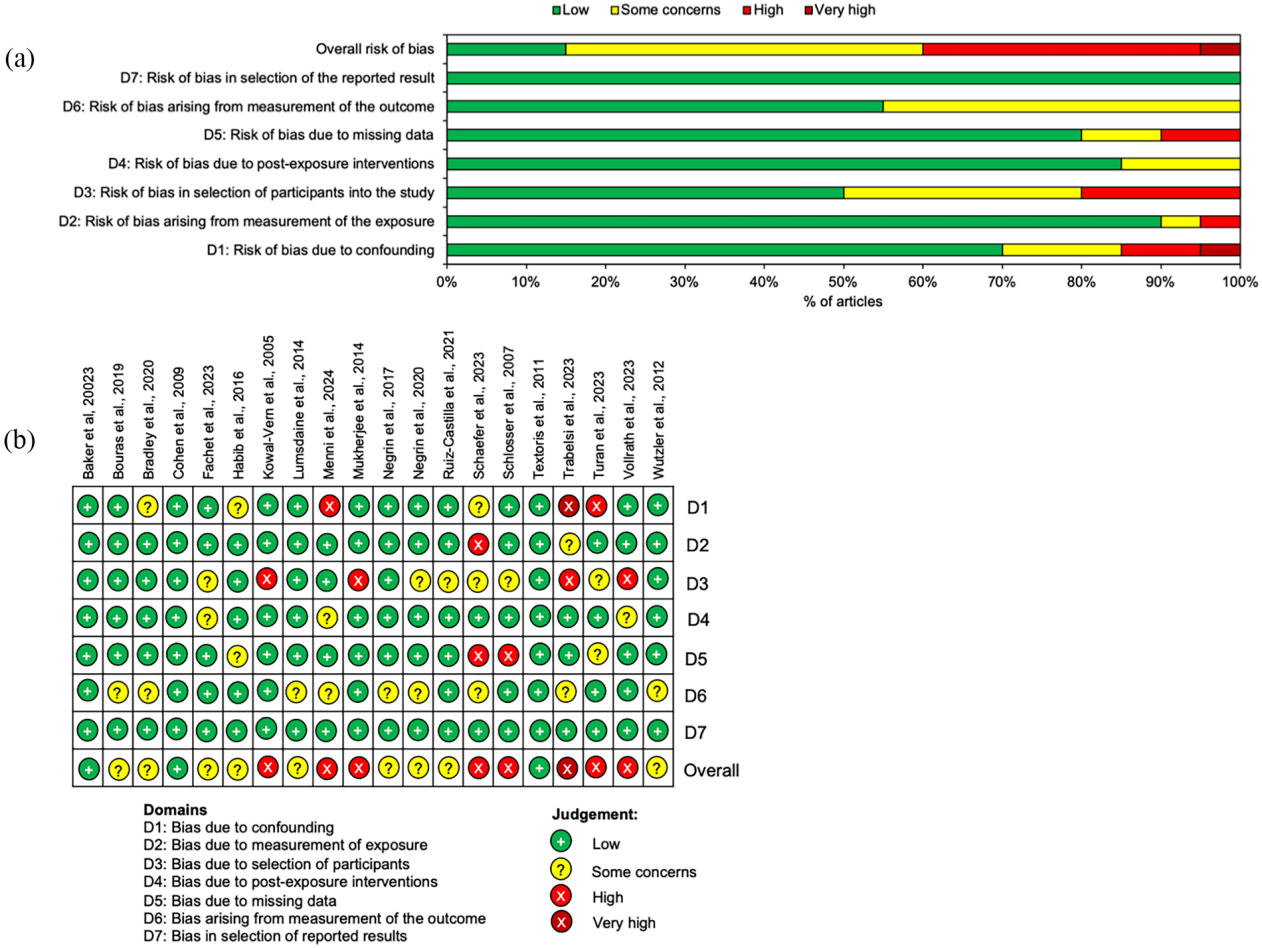
Risk of bias in the included studies. (a) Summary chart to show the risk of bias across the seven domains in all studies. (b) Diagram to show risk of bias in individual studies.

The ROBINS-E tool was selected to assess the risk of bias, estimating the effect of an exposure (i.e. pneumonia) on an outcome (i.e. blood-based biomarkers) in observational studies.^35^ The risk of bias analysis demonstrated some concerns to very high risk of bias in 85% of the studies (Figure 2(a)). As eight of the studies were judged as having an overall very high or high risk of bias and nine had some concerns, the overall judgement across the 20 studies was that of very high risk of bias. Half of the studies had some concerns or high risk of bias occurring in domain three (risk of bias in selection of participants into the study) and 45% of studies had some concerns of bias in domain six (risk of bias arising from measurement of the outcome; Figure 2(b)).

### Incidence of pneumonia

**Figure 3.**
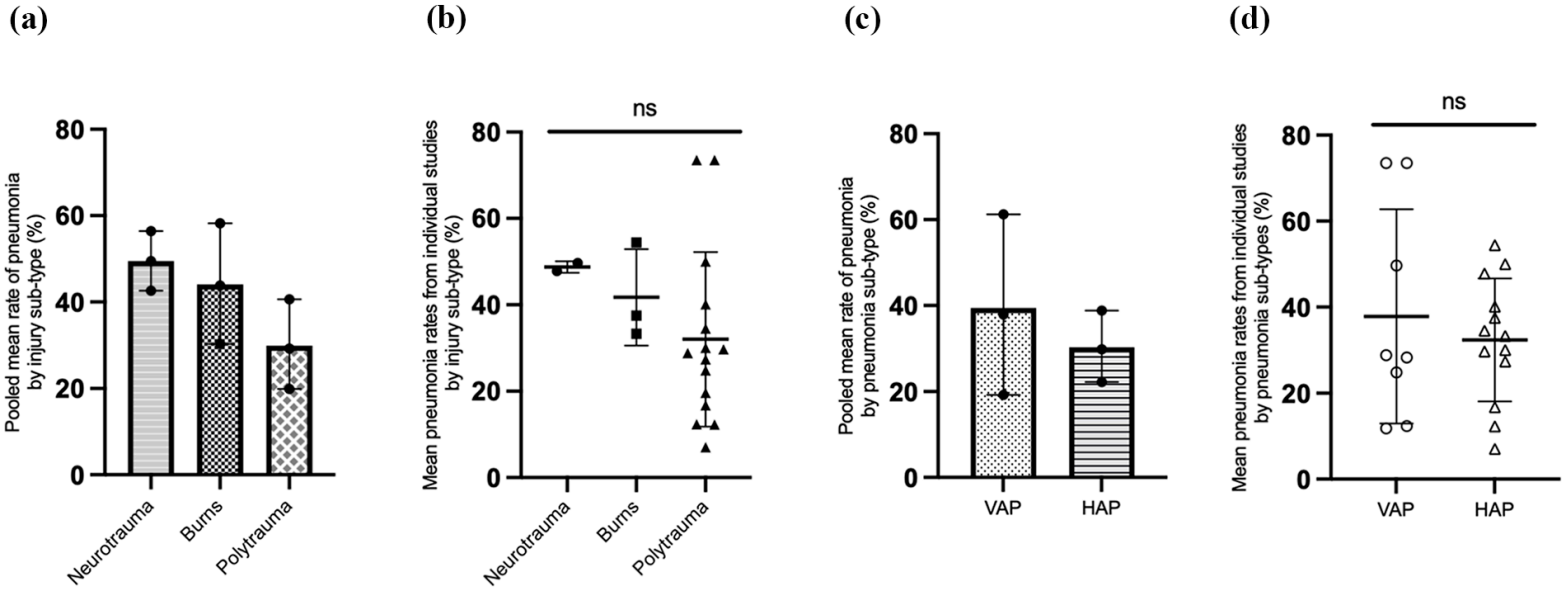
The incidence of pneumonia categorised by injury and pneumonia type. (a) Pooled mean rates of pneumonia by injury subtype; (b) Mean values of pneumonia rates reported by individual studies, according to injury subtype; (c) Pooled mean rates of pneumonia by pneumonia subtype; (d) Mean values of pneumonia rates reported by individual studies, according to pneumonia subtype. Figure 3(b) and (d) demonstrate a complementary analysis and should be interpreted with caution as the studies are not weighted. VAP: ventilator acquired pneumonia; HAP: hospital acquired pneumonia; ns: not significant.

The incidence of pneumonia varied between studies and the different types of trauma (Figure 3(a)). Two studies reported the incidence of pneumonia after neurotrauma, with a pooled mean pneumonia rate of 49.5% (42.6–56.4%). The pooled mean rate of pneumonia after burns and polytrauma was 43.8% (30.3–58.2%) and 29.2% (19.9–40.6%) respectively. An exploratory observation of the mean values of pneumonia rates reported in each study via one-way ANOVA with Bonferroni correction did not show any significant differences in pneumonia between injury sub-types (*p* = 0.42; Figure 3(b)).

The pooled mean rates of VAP (37.9% (19.2–61.2%)) were higher than the pooled mean rates of HAP (29.8% (22.2–38.8%); Figure 3(c)). An exploratory observation of the mean values of VAP and HAP rates reported in each study did not show any significant differences (unpaired student’s *t*-test) in pneumonia between pneumonia sub-types (*p* = 0.53; Figure 3(d)).

### Biomarker analysis

A total of 70 different biomarkers were evaluated across the 20 studies. At the timepoint of hospital admission there were 45 reported biomarkers. Eleven of the admission biomarkers were reported in two or more studies and therefore possible for meta-analysis (Table 2), with a further 34 biomarkers were reported in one study only (Supplemental File 2; Table 1). During hospitalisation there were 53 reported biomarkers, with nine possible for meta-analysis (Table 3) and 44 biomarkers reported in one study only (Supplemental File 2; Table 2).

**Table 2.** Meta-analysis for the effect of pneumonia on biomarkers reported at hospital admission.

Biomarker	SMD	Lower limit	Upper limit	*p*-value	Sample size (pneumonia)	Sample size (no-pneumonia)	*k*	*I*^2^ (%)	*Q*	Tau^2^	Egger *p*-value
CYFRA21-1	0.53	0.19	0.86	0.002**	50	124	2	0	0.12	0	N/A
Leucocytes	0.28	0.05	0.50	0.01**	130	214	2	0	0	0	N/A
IL-6	1.41	0.04	2.77	0.04*	85	276	4	95.05	60.55	1.82	0.44
Creatinine	−0.31	−0.64	0.02	0.06	49	100	2	0	0.93	0	N/A
Lymphocytes	−0.16	−0.41	0.09	0.21	111	180	3	0	0.28	0	0.22
IL-10	1.49	−1.22	4.20	0.28	59	247	2	98.5	65.09	3.76	N/A
sRAGE	−0.14	−0.47	0.19	0.40	50	124	2	0	0.40	0	N/A
NLR	−0.19	−0.67	0.29	0.44	22	90	2	0	0.21	0	N/A
CRP	−0.09	−0.34	0.16	0.47	133	119	3	0	1.96	0	0.04
Haemoglobin	−0.02	−0.28	0.23	0.85	81	230	3	0	0.34	0	0.46
Neutrophils	−0.04	−0.59	0.52	0.90	111	180	3	68.19	6.29	0.16	0.23

Results of meta-analysis using random effects model and SMD when biomarkers are reported in two or more studies at hospital admission.

CYFRA21-1: cytokeratin fragment 21-1; IL: interleukin; sRAGE: soluble receptor for advanced glycation-end products; NLR: neutrophil to lymphocyte ratio; CRP: C-reactive protein; SMD: standardised mean difference.

Egger test *p* value for publication bias only possible when *k* > 2, when *k* ⩽ 2 result reported as N/A.

* *p* > 0.05, **p > 0.01.

**Table 3.** Meta-analysis for effect of pneumonia on biomarkers during hospitalisation, reported by timepoint.

Biomarker	Time point	SMD	LL	UL	*p*	Sample size (pneumonia)	Sample size (no-pneumonia)	*k*	*I* ^2^	*Q*	Tau^2^	Egger test
CRP												
	At diagnosis	0.49	−0.16	1.13	0.14	36	13	1	N/A	N/A	N/A	N/A
	Day 1–2	0.46	−0.61	1.52	0.40	7	7	1	N/A	N/A	N/A	N/A
	Day 3	0.83	−0.28	1.93	0.14	103	113	2	89.33	1.00	0.57	N/A
	Day 4	1.87	1.51	2.24	>0.001***	67	100	1	N/A	N/A	N/A	N/A
	Day 5	1.38	1.03	1.72	>0.001***	67	100	1	N/A	N/A	N/A	N/A
	Day 6	0.74	0.42	1.06	>0.001***	67	100	1	N/A	N/A	N/A	N/A
	Day 7	0.87	0.12	1.63	0.02*	138	126	2	86.06	7.18	0.26	N/A
	Day 8	0.22	−0.09	0.54	0.16	67	100	1	N/A	N/A	N/A	N/A
	Day 9	−0.09	−0.40	0.22	0.56	67	100	1	N/A	N/A	N/A	N/A
IL-6												
	Day 1	1.54	−0.85	3.92	0.21	47	210	2	96.02	25.13	2.85	N/A
	Day 2	0.21	−0.35	0.77	0.46	59	247	2	69.59	3.29	0.11	N/A
	Day 3	3.11	2.66	3.55	>0.001***	39	194	1	N/A	N/A	N/A	N/A
	Day 5	0.57	0.05	1.09	0.03*	20	53	1	N/A	N/A	N/A	N/A
	NR	0.99	0.28	1.71	0.01*	9	64	1	N/A	N/A	N/A	N/A
IL-10												
	Day 1	4.42	3.89	4.95	>0.001***	39	194	1	N/A	N/A	N/A	N/A
	Day 2	−0.83	−2.46	0.79	0.32	59	247	2	96.17	26.10	1.32	N/A
	Day 3	−0.29	−0.64	0.05	0.10	39	194	1	N/A	N/A	N/A	N/A
	Day 5	0.38	−0.14	0.90	0.15	20	53	1	N/A	N/A	N/A	N/A
CYFRA21-1												
	Day 2	0.43	0.10	0.76	0.01*	50	124	2	0	0.77	0	N/A
	Day 5	−0.03	−0.54	0.49	0.92	20	53	1	N/A	N/A	N/A	N/A
sRAGE												
	Day 2	0.16	−0.17	0.49	0.34	50	124	2	0	0.03	0	N/A
	Day 5	−0.08	−0.60	0.43	0.75	20	53	1	N/A	N/A	N/A	N/A
Lymphocytes	Day 7	−0.28	−0.76	0.19	0.24	22	90	2	0	0.45	0	N/A
Neutrophils	Day 7	0.15	−0.66	0.95	0.72	22	90	2	62.18	2.64	0.21	N/A
NLR	Day 7	0.98	−1.31	3.27	0.40	22	90	2	94.59	18.49	2.58	N/A
NOBC	Day 1	1.52	0.96	2.09	>0.001***	14	186	2	0	0.35	0	N/A

Results of meta-analysis using random effects model and SMD when biomarkers are reported in two or more studies at hospital admission. When one study reported for the remaining timepoints; meta-analysis not possible therefore reported as SMD and confidence interval calculation.

SMD: standardised mean difference; LL: lower limit; UL: upper limit; CRP: C-reactive protein; IL: interleukin; CYFRA21-1: cytokeratin fragment 21-1; sRAGE: soluble receptor for advanced glycation-end products; NLR: neutrophil to lymphocyte ratio; NOBC: neutrophil oxidative burst capacity.

Egger test *p* value for publication bias only possible when *k* > 2, therefore when *k* ⩽ 2 result reported as N/A.

* *p* > 0.05, **p > 0.01.

#### Biomarkers at hospital admission

Three of the 11 biomarkers demonstrated a statistically significant SMD at hospital admission. At admission IL-6 protein (SMD 1.41 (0.04–2.77), *p* = 0.04), CYFRA21-1 (0.53 (0.19–0.86), *p* = 0.002) and leucocytes (0.28 (0.05–0.50), *p* = 0.01) were higher in the patients who later developed pneumonia than those without pneumonia, whilst no significant difference was observed for the other biomarkers.

#### Biomarkers during hospitalisation

Nine biomarkers were meta-analysed across 11 time-points during hospitalisation (Table 3). During hospitalisation, patients with pneumonia had significantly higher IL-10 (4.42 (3.89–4.95), *p* > 0.001) and neutrophil oxidative burst capacity (1.52 (0.96–2.09), *p* > 0.001) at day 1 of hospitalisation, increased CYFRA21-1 at day 2 (0.43 (0.10–0.76), *p* = 0.01), increased IL-6 at day 3 (3.11 (2.66–3.55), *p* > 0.001) and day 5 (0.57 (0.05–1.09), *p* = 0.03) and higher CRP at day 4 (1.87 (1.51–2.24), *p* > 0.001), day 5 (1.38 (1.03–1.72), *p* > 0.001), day 6 (0.74 (0.42–1.06), *p* > 0.001) and day 7 (0.87 (0.12–1.63), *p* = 0.02).

Interleukin-6 (IL-6) and C-Reactive Protein (CRP) were the most frequently reported biomarkers across all timepoints. Due to low study numbers and high levels of heterogeneity across all biomarkers, the meta-analysis results for CRP and IL-6 have been combined with narrative synthesis.

##### C-reactive protein

**Figure 4.**
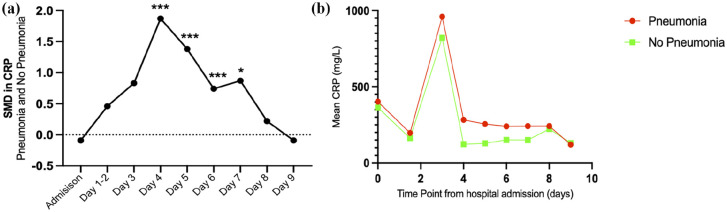
C-Reactive Protein (CRP) during hospitalisation in pneumonia and no pneumonia groups. (a) SMD in CRP between patients with pneumonia and without pneumonia during hospitalisation after major trauma to demonstrate trends over time. (b) Exploratory observation of the mean values of CRP reported in each study providing a representation of clinical metric; please note this is a complementary analysis and should be interpreted with caution as the studies are not weighted. *p > 0.05. ***p > 0.001.

CRP was the most frequently reported biomarker, reported in 6 of the 20 included studies and across 9 different timepoints during hospitalisation (Table 3). Assessment methods for CRP included electrochemiluminescence assays, nephelometry and routinely available clinical laboratory records. No significant differences in CRP were detected at admission baseline (SMD −0.009 (95% CI −0.34 to 0.16), *p* = 0.47) between patients with and without pneumonia. During hospitalisation, results demonstrated a statistically significant increase in CRP at day 4 (1.87 (1.51–2.24), *p* > 0.001), day 5 (1.38 (1.03–1.72), *p* > 0.001), day 6 (0.74 (0.42–1.06), *p* > 0.001) and day 7 ( 0.87 ( 0.12–1.63), *p* = 0.02) in patients with pneumonia (Figure 4(a)). Trends suggest that during hospitalisation, patients with pneumonia have higher levels of CRP, with a peak increase at day 3 (Figure 4(b)).

##### Interleukin-6

**Figure 5.**
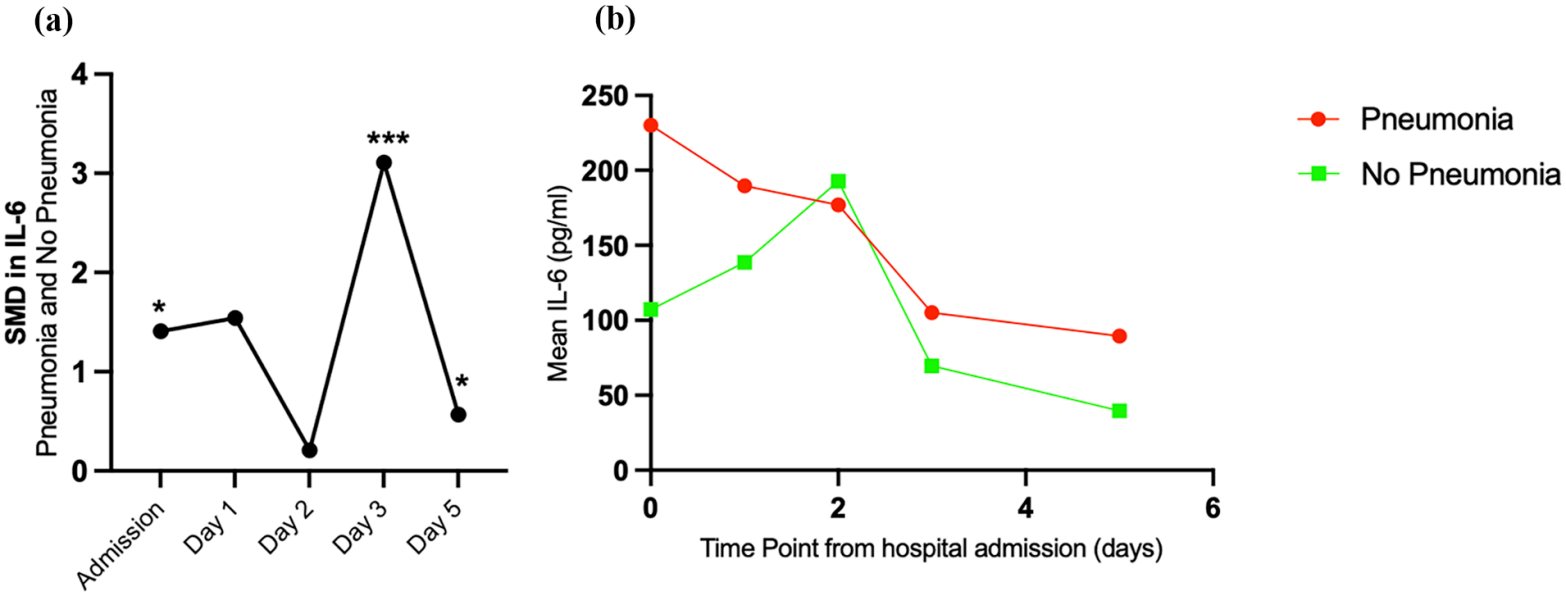
Interleukin-6 (IL-6) during hospitalisation in pneumonia and no pneumonia groups. (a) SMD in IL-6 between patients with pneumonia and without pneumonia during hospitalisation after major trauma to demonstrate trends over time. (b) Exploratory observation of the mean values of IL-6 reported in each study providing a representation of clinical metric; please note this is a complementary analysis and should be interpreted with caution as the studies are not weighted. *p > 0.05. ***p > 0.001.

IL-6 was reported in five studies across four known timepoints during hospitalisation. Assessment methods for IL-6 across studies included Luminex, ELISA and Pico Scan densitometer within a breadth of injury sub-types (polytrauma, neurotrauma and burns). Patients who developed pneumonia after major trauma presented with higher IL-6 protein levels at admission (1.41 (0.04–2.77), *p* = 0.04), compared with patients without pneumonia. There were no significant differences between groups at day 1 and 2 of hospitalisation, with high heterogeneity at these time points (*I*^2^ 96.02% and 69.59%, respectively). The greatest difference in IL-6 between pneumonia and no-pneumonia patients was seen at day 3 during hospitalisation (3.11 (2.66–3.55), *p* > 0.001; Figure 5(a)). Trends suggested that trauma patients with pneumonia have higher levels of IL-6 compared to those without pneumonia at hospital admission, day 1, day 3 and day 5 (Figure 5(b)).

### Clinical diagnosis of pneumonia

The reported clinical diagnostic methods for pneumonia varied across studies, identifying heterogeneity in the biomarker reference standards. Nine different categories of diagnostic tools were recorded across the 20 studies; each study utilising 0–8 of the categories for a diagnosis of pneumonia (Table 4). The most frequently used tools were new or progressive infiltrates on chest X-ray (*n* = 18), change in temperature (*n* = 14), change in sputum characteristics (*n* = 14) and tracheal culture (*n* = 14). Five of the studies reported the planned timepoint for pneumonia assessment, with most stating that diagnosis had to occur 48-h after hospital admission. Nine studies reported the time of pneumonia diagnosis, with an overall mean onset of day 5 after hospitalisation (range day 2–day 8).

**Table 4.** Diagnostic methods for pneumonia.

Study (Author, year (reference))	Time of pneumonia assessment	Temperature	WBC	Sputum	Clinical symptoms or bedside assessment	Oxygen levels	Chest imaging	Tracheal culture	Guideline reference	Senior clinician diagnosis	Total
Baker et al., 2003^40^	NR	x	x	x		x	x	x			6
Bouras et al., 2019^41^	⩾48 h	x	x	x			x	x			5
Bradley et al., 2020^42^	⩾48 h						x	x			2
Cohen et al., 2009^43^	NR						x	x	x		3
Fachet et al., 2023^44^	NR						x	x			2
Habib et al., 2015^36^	NR	x	x	x			x	x			5
Kowal-Vern et al., 2005^45^	NR	x					x	x			3
Lumsdaine et al., 2014^46^	NR	x	x	x		x	x				5
Menni et al., 2024^47^	⩾48 h	x	x	x		x	x	x			6
Mukherjee et al., 2014^48^	⩾48 h	x	x	x	x	x	x	x	x		8
Negrin et al., 2017^37^	NR	x	x	x	x		x	x			6
Negrin et al., 2020^49^	NR	x	x	x	x		x				5
Ruiz-Castilla et al., 2021^50^	NR	x	x	x		x	x		x		6
Schaefer et al., 2023^38^	NR										0
Schlosser et al., 2007^51^	Within 1 week			x	x		x	x	x		5
Textoris et al., 2011^52^	NR	x	x	x			x	x		x	6
Trabelsi et al., 2023^53^	NR								x		1
Turan et al., 2023^54^	NR	x		x	x		x				4
Vollrath et al., 2023^55^	NR	x	x	x	x		x	x			6
Wutzler et al., 2012^39^	NR	x	x	x			x	x			5
**Total**	5	14	12	14	6	5	18	14	5	1	

WBC: white blood cell count.

Clinical symptoms or examination included features such as cough, tachypnoea, dyspnoea, auscultation or percussion.

## Discussion

This systematic review was conducted to synthesise the evidence for blood-based biomarkers for pneumonia in patients acutely hospitalised after major trauma. Twenty studies consisting of 4316 patients were included in the analysis, with an overall pneumonia rate of 32.7%. Trends suggested that incidence rates of pneumonia attributed to VAP were higher than that associated with HAP and that the rates of pneumonia were higher in neurotrauma patients compared to other injuries. Meta-analysis identified a statistically significant increase in IL-6, CYFRA21-1 and leucocyte count at hospital admission, therefore suggesting their potential as early predictive markers for pneumonia after major trauma. During the first week of hospitalisation after major trauma, patients with pneumonia had significantly higher IL-10, neutrophil oxidative burst capacity, CYFRA21-1, IL-6 and CRP. Of the 70 biomarkers identified, IL-6 and CRP were the most frequently studied, with overall trends suggesting higher protein levels in patients who developed pneumonia after major trauma.

### Pneumonia rates after major trauma

Pneumonia was reported in approximately one-third of patients after major trauma (32.7% (23.5%–43.4%)), yet varied across injury and pneumonia sub-types. The overall incidence of pneumonia is similar to previously published studies, yet there is known variability across the literature.^8,17^ Over 90% of patients included in the review were categorised as polytrauma, with an average pneumonia rate for this sub-group of 29.2% (19.9%–40.6%). Polytrauma is a heterogenous term including a multitude of different injury types and severities which may influence the risk of pneumonia.^1^ For example, patients with thoracic trauma are four times more likely to develop pneumonia than those without such injuries and patients with higher Injury Severity Scores also present greater risk.^8,17^ There are also numerous variations in clinical management after major trauma which may influence the susceptibility to pneumonia; including immobilisation, mechanical ventilation, and repeated surgical procedures, as well as the patient’s pre-admission health status and severity of illness.^7,56,57^ Such details were not collated as part of this review due to the discrepancies in reporting across studies and therefore could not be evaluated in analysis.

The inconsistencies in pneumonia rates may also be explained by the variation in clinical diagnostic methods for pneumonia across studies. Nine different categories of diagnostic tools were recorded across the 20 studies; each study utilising 0–8 of the categories for a diagnosis of pneumonia. Chest imaging was the most frequently used tool, recorded in 90% of studies. However, radiological diagnosis of pneumonia after major trauma is notoriously challenging; with limitations in adequate positioning due to injury restrictions and the overlap of many common pathologies which may also present with infiltrates after major trauma such as pulmonary contusions, blast lung injury or smoke inhalation.^58^ Traditionally pneumonia diagnosis relies on a combination of clinical, radiographic and laboratory criteria.^7,8,19^ Many of the common diagnostic methods are challenging in the major trauma patient, facing subsequent risk of inaccurate reporting and variation in biomarker reference standards.^17,56,59^ The heterogeneity in pneumonia reporting requires consideration in the interpretation of these results, with further studies required to determine appropriate pneumonia reference standards.

The incidence rates of pneumonia attributable to VAP were higher than HAP (37.9% (19.2%–61.2%) vs 29.8 (22.2%–38.8%)). VAP is a pneumonia occurring at least 48-h after tracheal intubation and is the most prevalent nosocomial infection in the ICU.^7^ Major trauma patients may require tracheal intubation during emergency resuscitation due to respiratory or airway compromise or if requiring general anaesthesia.^60^ Such patients represent a major trauma cohort who are critically injured, commonly presenting with high ISS and low GCS and therefore have increased susceptibility to infection.^60^ Although a life-saving intervention, the endotracheal tube (ETT) itself contributes to the risk of VAP.^7,61^ The ETT is a source of pathogenic colonisation with the development of a biofilm within hours of intubation and pooling reservoirs of secretions around it’s protective cuff.^61^ As the ETT breaches the normal anatomical barrier of the upper airway it prevents epiglottis closure, diminishes upper airway reflexes of coughing and provides pathogens direct access to the respiratory tract.^61^

### Biomarkers for pneumonia after major trauma at hospital admission

At hospital admission, there were 45 reported biomarkers for pneumonia across the 20 included studies, with 11 reported in two or more studies and therefore possible for meta-analysis. At admission IL-6 protein (SMD 1.41 (0.04–2.77), *p* = 0.04), CYFRA21-1 (0.53 (0.19–0.86), *p* = 0.002) and leucocytes (0.28 (0.05–0.50), *p* = 0.01) were higher in the patients who later developed pneumonia than those without pneumonia. The greatest SMD was seen in IL-6, which was reported in four studies with low publication bias (Eggers test *p* = 0.44), yet with notably high heterogeneity (*I*^2^ 95.05%, *Q* 60.55%). Although CYFRA21-1 and leucocyte count demonstrated low heterogeneity (*I*^2^ >0.5% and *Q* 0% for both studies), they were only reported in two studies with overall low sample sizes. Although these three biomarkers may offer potential as early predictive markers for pneumonia after major trauma, the low study numbers and high heterogeneity means that results should be interpreted with caution.

### Biomarkers for pneumonia after major trauma during hospitalisation

During hospitalisation there were 53 reported biomarkers, with nine possible for meta-analysis across 11 time-points. Five of the biomarkers were statistically significant, demonstrating higher biomarker levels in patients who developed pneumonia after major trauma than those who did not develop pneumonia. At day 1 of hospitalisation, patients who developed pneumonia had significantly higher IL-10 (4.42 (3.89–4.95), *p* > 0.001) and neutrophil oxidative burst capacity (1.52 (0.96–2.09), *p* > 0.001), however this result is based on low study numbers with small sample sizes. At day 2 of hospitalisation, pneumonia patients had increased CYFRA21-1 (0.43 (0.10–0.76), *p* = 0.01), reported in just two studies although with low heterogeneity. At day 3 and day 5, there was a statistically significant increase in IL-6 levels seen in patients with pneumonia (3.11 (2.66–3.55), *p* > 0.001 and 0.57 (0.05–1.09), *p* = 0.03, respectively), yet each reported in one study alone. Lastly, there was a statistically significant increase in CRP between day 4 and day 7 after hospitalisation in those who developed pneumonia (day 4: 1.87 (1.51–2.24), *p* > 0.001; day 5:1.38 (1.03–1.72), *p* > 0.001; day 6:0.74 (0.42–1.06), *p* > 0.001 and, day 7:0.87 (0.12–1.63), *p* = 0.02) yet results are again confounded by low study numbers and sample sizes. Although these biomarkers may serve as potential indictors for pneumonia, their clinical application is confounded by the heterogeneity in pneumonia reporting. As the time of pneumonia diagnosis varied between studies (mean onset at day 5 (range day 2–day 8)), with less than half of the studies reporting the time point of clinical onset, the utility of the reported biomarkers is currently limited. Although there is association between pneumonia after major trauma and increased levels of IL-10, neutrophil oxidative burst capacity, CYFRA21-1, IL-6 and CRP during hospitalisation, their accuracy and clinical relevance as a predictive, diagnostic or prognostic tool is yet to be determined.

### IL-6 as a biomarker for pneumonia after major trauma

The results of the meta-analysis identified a statistically significant SMD in IL-6 between trauma patients with pneumonia and those without pneumonia at hospital admission (1.41 (CI 0.04–2.77), *p* = 0.04) and at day 3 during hospitalisation (3.11 (2.66–3.55), *p* > 0.001). IL-6 is an inflammatory cytokine, produced in response to invading pathogens.^62^ Homeostatic levels of IL-6 provide a well-coordinated and regulated innate immune response that is essential to control viral infections.^63^ However, overexpression of IL-6 can have negative consequences, leading to viral persistence, immune dysfunction and severe systemic inflammatory response through cytokine storming.^62,63^ IL-6 is released in response to a multitude of inflammatory stimuli, including trauma, surgery, multi-organ failure and critical illness.^64^ Furthermore, as IL-6 is produced locally by type-2 epithelial cells and alveolar macrophages, it also increases in response to other causes of respiratory system inflammation, such as acute respiratory distress syndrome or biotrauma induced by mechanical ventilation.^64^ Although the results highlight IL-6 as a potential predictive biomarker for pneumonia after major trauma, further studies are required to determine its specificity, and to define a clinically meaningful metric that distinguishes pneumonia from other causes of local and systemic inflammation after trauma.

The methods and time points of IL-6 measurement varied between studies. Although IL-6 assays are relatively inexpensive and easy to perform, they are not commonly available in all institutions.^64^ Specific methods and timepoints of IL-6 measurement therefore require further exploration and standardisation, in order to improve clinical application. The timing of measurement is imperative after major trauma, as IL-6 concentrations are known to vary over time, peaking at 24-h after injury.^64^ The magnitude of IL-6 elevation is also known to correlate with the extent of tissue trauma and injury severity, which is associated with adverse outcomes, including mortality.^64^ Due to the heterogeneity between study populations and inconsistencies in reporting, studies were grouped based on injury sub-type rather than severity, and differences in clinical management and outcomes could not be evaluated in analysis. Further research is required to define specific IL-6 assessment methods for pneumonia after major trauma and to determine their clinical application and prognostication through prospective, sequential evaluation.

### CRP as a biomarker for pneumonia after major trauma

Results also identified high levels of CRP in association with pneumonia after major trauma. There were no significant differences in CRP detected at admission baseline (−0.009 (−0.34 to 0.16), *p* = 0.47), yet despite low study numbers, the results during hospitalisation demonstrated statistically significant SMD between day 4 and day 7. CRP is an acute-phase reactant protein synthesised in the liver, that rises in response to any form of inflammation, including infection, trauma or surgery.^65^ CRP is often preceded by a rise in IL-6, with studies suggesting a simultaneous systemic inflammatory response within 12-h.^64^ CRP is often a surrogate marker for IL-6 as it is a more readily available measure of inflammation.^64^ Traumatic injury leads to a rapid and complex immune response, characterised by systemic inflammation and immune activation, alongside compensatory immune suppression.^56,66^ CRP symbiotically rises in response to trauma, with a sustained response for several days that is uniform after any injury.^66^ Although a pragmatic measure that is readily available in practice, as CRP is a non-specific marker of inflammation, the utility of CRP as a biomarker for pneumonia after trauma is questionable and likely to be reliant upon repeated measures in conjunction with clinical features. Alterative assessment methods of CRP may offer a more accurate understanding of the localised inflammatory response associated with pneumonia, such as bronchoscopy or exhaled breath condensate. However, such techniques were not evaluated within the scope of this review. Nonetheless, CRP as a blood-based biomarker has been associated with other respiratory pathologies; including community acquired pneumonia and COVID-19.^26,67^ Results suggest that CRP may be a useful indicator of pneumonia during hospitalisation after major trauma. However, due to low study numbers and high heterogeneity, results should be interpreted with caution. Further studies are required to validate the use of CRP as a potential biomarker after major trauma and identify the appropriate cut-off level specific to pneumonia diagnosis.

### Limitations

This systematic review has some limitations. Firstly, only studies published in English language were selected due to the time and cost associated with translation services, therefore imposing potential language bias. However, as language restriction was applied at the stage of study selection, rather than study searching, the number of studies excluded due to language was recorded. In this systematic review, only 4 of the 4124 excluded studies were due to language and therefore the effects of language bias in this study are minor. Furthermore, as the search strategy only included published studies from peer-reviewed journals, the exclusion of grey literature may also introduce publication bias. A further limitation is the high heterogeneity across all included studies; with large variation in the biomarkers investigated, the methods and time points for biomarker assessment, the clinical reference standards for pneumonia diagnosis and patient characteristics including injury sub-type. Although heterogeneity in clinical characteristics introduces potential confounders, it is a pragmatic investigation of the complexities of a major trauma population and representative of the clinical challenges faced when diagnosing pneumonia. Despite the high heterogeneity, a meta-analysis using SMD was possible for 12 of the 70 identified biomarkers and performed using a random-effects model, which assumes that the true effect could vary from study to study. The results were also limited by the low number of studies in each of the meta-analyses. Furthermore, 85% of all included studies demonstrated some concerns to very high risk of bias and therefore results are to be interpreted with caution.

## Conclusion

This systematic review investigated blood-based biomarkers for pneumonia after major trauma. Affecting approximately one-third of patients, pneumonia after major trauma is a major cause of mortality and morbidity in all age groups globally and therefore requires urgent attention to improve diagnostic capability. The heterogeneity across trauma populations, clinical diagnostic tools for pneumonia and biomarker testing methods introduces potential for confounding factors and moderate to very high risk of bias. However, the results of this systematic review identify potential biomarkers for pneumonia at hospital admission and within the first week of hospitalisation in major trauma patients. Further high-quality studies are required to confirm the results of this systematic review and to define specific biomarker assessment methods and time points. Future studies should also consider the prognostic value of biomarkers for pneumonia after trauma, assessing mortality, morbidity, and its impact on mitigating antibiotic pressure in the intensive care unit.

## Supplemental Material

sj-docx-1-inc-10.1177_17511437251344068 – Supplemental material for Biomarkers for pneumonia after major trauma: A systematic review and meta-analysis Supplemental material, sj-docx-1-inc-10.1177_17511437251344068 for Biomarkers for pneumonia after major trauma: A systematic review and meta-analysis by Fiona Howroyd, Amanda Veiga Sardeli, Fang Gao Smith, Tonny Veenith, Niharika A Duggal and Zubair Ahmed in Journal of the Intensive Care Society

sj-docx-2-inc-10.1177_17511437251344068 – Supplemental material for Biomarkers for pneumonia after major trauma: A systematic review and meta-analysis Supplemental material, sj-docx-2-inc-10.1177_17511437251344068 for Biomarkers for pneumonia after major trauma: A systematic review and meta-analysis by Fiona Howroyd, Amanda Veiga Sardeli, Fang Gao Smith, Tonny Veenith, Niharika A Duggal and Zubair Ahmed in Journal of the Intensive Care Society

sj-docx-3-inc-10.1177_17511437251344068 – Supplemental material for Biomarkers for pneumonia after major trauma: A systematic review and meta-analysis Supplemental material, sj-docx-3-inc-10.1177_17511437251344068 for Biomarkers for pneumonia after major trauma: A systematic review and meta-analysis by Fiona Howroyd, Amanda Veiga Sardeli, Fang Gao Smith, Tonny Veenith, Niharika A Duggal and Zubair Ahmed in Journal of the Intensive Care Society
