# Supplementary material for: Biomarkers for pneumonia after major trauma: A systematic review and meta-analysis
Source: J Intensive Care Soc. 2025 Jun 13;27(1):66–81. doi: 10.1177/17511437251344068 (PMC12165960; doi:10.1177/17511437251344068)

**Supplementary File 1**

**Syntax:**

Preliminary scoping searches identified predominantly observational cohort studies. The PECO syntax has therefore been used to determine key words and inclusion criteria;

- Population – humans, adults (aged 18 years or above), hospitalised after major trauma
- Exposure – diagnosed with pneumonia after hospitalisation with major trauma
- Control – no pneumonia after hospitalisation with major trauma
- Outcome – any blood biomarker

**Question:**

To identify blood-based biomarkers associated with pneumonia after major trauma

**Table 1:** Keywords, synonyms, heading (MeSH) and entry terms for literatrure search

| **Keyword** | **Entry terms search within Abstract and Title** |
| --- | --- |
| **Pneumonia** | ‘Pneumonia*’ OR ‘pneumonitis’ OR ‘Pneumonitides’ OR ‘Pulmonary Inflammation’ OR ‘Inflammation, Pulmonary’ OR ’Lung Inflammation’ OR ‘Inflammation, Lung’ OR ‘Respiratory tract infection’ OR ‘Bronchopneumonia’ OR ‘Lobar Pneumonia’ |
| **Biomarkers** | ‘biomarker*‘ OR ‘Marker* Biologic*‘ OR ‘Biologic* Marker*’ OR ‘Marker* Immun*’ OR ‘Immun* Marker*’ OR ‘Serum Marker*’ OR ‘Marker* Serum’ OR ‘Surrogate End point*’ OR ‘Endpoint* Surrogate*’ OR ‘Surrogate endpoint’ OR ‘End Point, Surrogate’ OR ‘Marker* Clinical’ OR ‘Clinical Marker*’ OR ‘Viral Marker*’ OR ‘Marker* Viral’ OR ‘biochemical marker*’ OR ‘marker biochemical*’ OR ‘laboratory marker*’ OR ‘marker* laboratory’ OR ‘surrogate marker*’ OR ‘marker* surrogate’ OR ‘blood marker*’ OR ‘marker* blood’ |
| **Major Trauma** | ‘multiple trauma’ OR ‘wound* injur*’ OR ‘injur* wound*’ OR ‘physical trauma’ OR ‘trauma physical’ OR ‘trauma*’ OR ‘injur*’ OR ‘wound*’ OR ‘multiple trauma*’ OR ‘wound* multiple’ OR injur* multiple’ OR ‘multiple injur*’ OR ‘major trauma’ OR ‘trauma major’ OR ‘fracture* multiple’ OR ‘multiple fracture*’ |

**Example search strategy using electronic databases:**


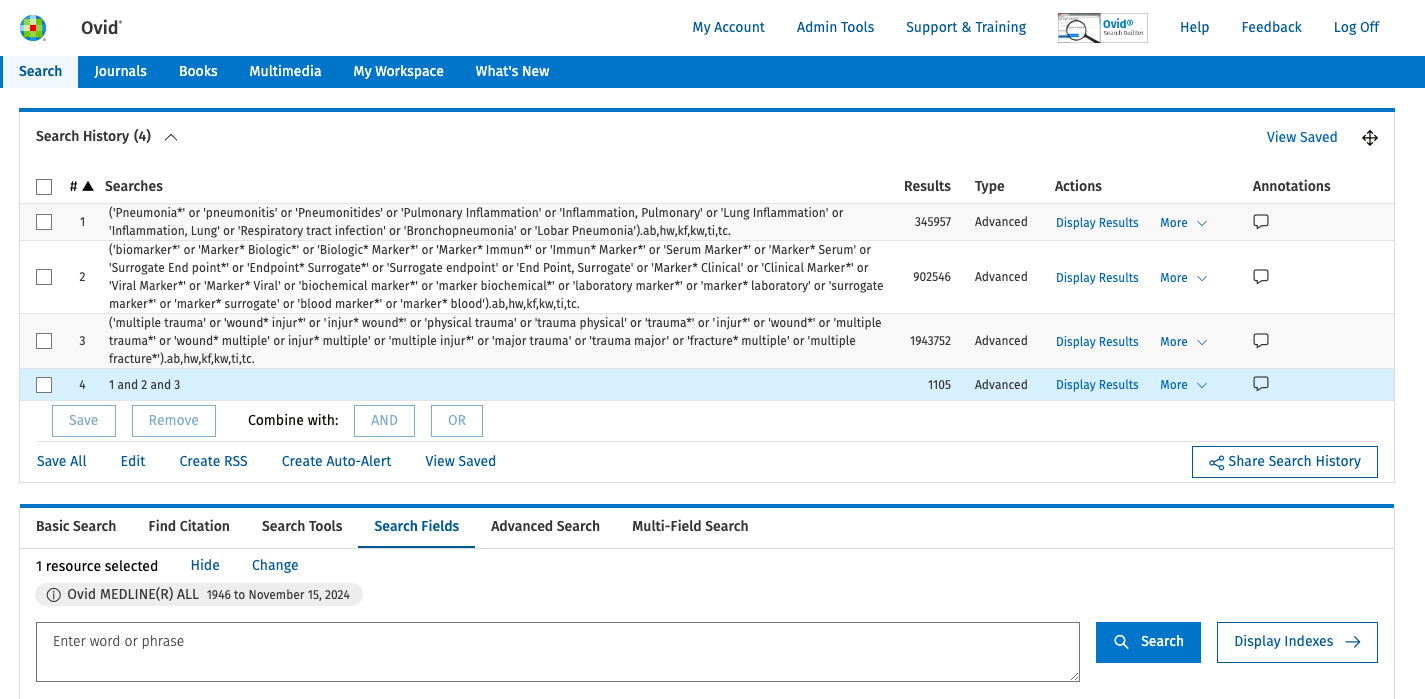


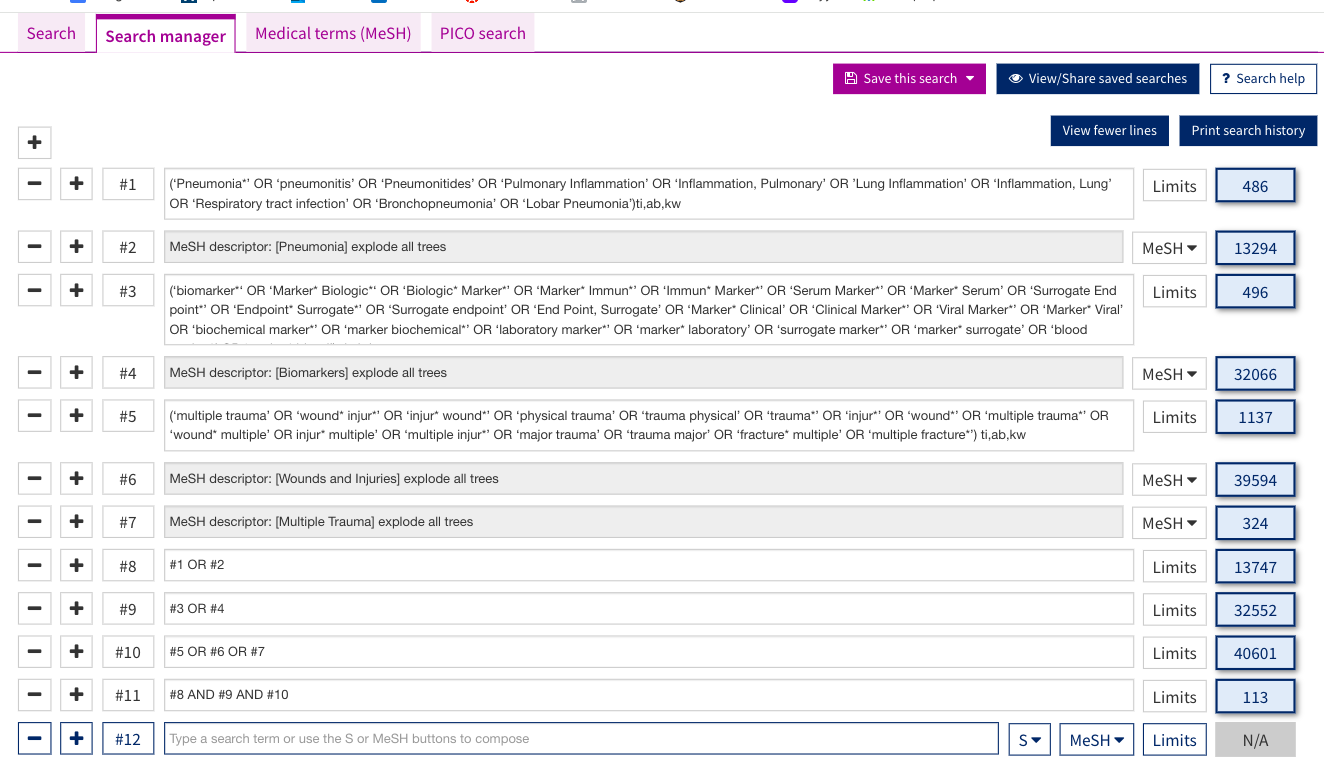

Supplement: sj-docx-1-inc-10.1177_17511437251344068 – Supplemental material for Biomarkers for pneumonia after major trauma: A systematic review and meta-analysis [file sj-docx-1-inc-10.1177_17511437251344068.docx]
