# Supplementary material for: Biomarkers for pneumonia after major trauma: A systematic review and meta-analysis
Source: J Intensive Care Soc. 2025 Jun 13;27(1):66–81. doi: 10.1177/17511437251344068 (PMC12165960; doi:10.1177/17511437251344068)
Supplement: sj-docx-2-inc-10.1177_17511437251344068 – Supplemental material for Biomarkers for pneumonia after major trauma: A systematic review and meta-analysis [file sj-docx-2-inc-10.1177_17511437251344068.docx]

**Supplementary File 2**

**Table 1:** SMD and confidence intervals for admission biomarkers reported in 1 study only

When one study reported; meta-analysis not possible therefore reported as SMD and confidence interval calculation. **Abbreviations:** IL = interleukin; PAI-1 = plasma activator inhibitor 1, SELENBP1 = selenium-binding protein 1, CRP = C-reactive protein.; TREM = triggering receptor expressed on myeloid cells, CC16 = club cell protein 16; SP-D = surfactant protein D; TNF = tumor necrosis factor ; KL-6/MUC1 = Krebs von den Lungen 6/Mucin 1; nCD64 = neutrophil cluster of differentiation 64; PaC02 = partial pressure of carbon dioxide; SMD = standardised mean difference; VAP = ventilator acquired pneumonia; NR = not recorded

| Biomarker | Effect Size (SMD) | Lower Limit | Upper Limit | *p* | Sample size  Pneumonia | Sample size  No-Pneumonia |
| --- | --- | --- | --- | --- | --- | --- |
| Fibrinogen | 1.26 | 0.88 | 1.64 | >0.001*** | 41 | 124 |
| Gene Expression AHSP | 0.63 | 0.27 | 0.99 | >0.001*** | 41 | 124 |
| Gene Expression ALAS2 | -0.96 | -1.32 | -0.58 | >0.001*** | 41 | 124 |
| Gene Expression IL-1B | 1.06 | 0.68 | 1.43 | >0.001*** | 41 | 124 |
| Gene Expression IL-1RN | 1.05 | 0.68 | 1.42 | >0.001*** | 41 | 124 |
| Gene Expression IL-6R | -1.36 | -1.74 | -0.98 | >0.001*** | 41 | 124 |
| Gene Expression PCSK1 | -1.70 | -2.09 | -1.30 | >0.001*** | 41 | 124 |
| Gene Expression PPBP | -1.54 | -1.93 | -1.15 | >0.001*** | 41 | 124 |
| PAI-1 | 2.32 | 1.48 | 3.17 | >0.001*** | 12 | 28 |
| SELENBP-1 | 1.07 | 0.56 | 1.58 | >0.001*** | 37 | 31 |
| Soluble Endothelial Plasma Protein-C | 1.03 | 0.43 | 1.62 | >0.001*** | 17 | 42 |
| Soluble Thrombomodulin | 4.61 | 3.61 | 5.62 | >0.001*** | 17 | 42 |
| Cortisol total / CRP ratio | -0.47 | -0.77 | -0.18 | 0.002** | 89 | 90 |
| Gene Expression TREM | -0.58 | -0.94 | -0.22 | 0.002** | 41 | 124 |
| CC16 | 0.63 | 0.19 | 1.06 | 0.005** | 30 | 71 |
| Blood cortisol total concentration | 0.30 | 0.01 | 0.59 | 0.04* | 89 | 90 |
| SP-D | -0.44 | -0.88 | -0.01 | 0.04* | 30 | 71 |
| Cortisol free / CRP ratio | 0.29 | 0.003 | 0.59 | 0.05* | 89 | 90 |
| Gene Expression TNF | 0.32 | -0.03 | 0.68 | 0.08 | 41 | 124 |
| Blood cortisol free concentration | 0.22 | -0.07 | 0.52 | 0.13 | 89 | 90 |
| KL-6/MUC1 | 0.31 | -0.11 | 0.74 | 0.15 | 30 | 71 |
| Transcortin | 0.21 | -0.08 | 0.51 | 0.16 | 89 | 90 |
| Pentraxin 3 | -0.35 | -0.86 | 0.17 | 0.19 | 20 | 53 |
| Plasma Lactate | -0.22 | -0.57 | 0.14 | 0.23 | 41 | 124 |
| Lactate | 0.40 | -1.67 | 0.47 | 0.27 | 7 | 7 |
| Gene Expression SPARC | 0.15 | -0.21 | 0.50 | 0.41 | 41 | 124 |
| Platelet Count | -0.13 | -0.45 | 0.20 | 0.44 | 49 | 140 |
| nCD64 | -0.22 | -0.86 | 0.41 | 0.49 | 36 | 13 |
| PaC02 | -0.04 | -0.33 | 0.25 | 0.78 | 89 | 90 |
| Albumin | 0.06 | -0.78 | 0.91 | 0.88 | 8 | 16 |
| Angiopoetin-2 | 0.04 | -0.47 | 0.55 | 0.88 | 20 | 53 |
| Bilirubin | 0 | -0.85 | 0.85 | 1.00 | 8 | 16 |
| Procalcitonin | 0 | -0.63 | 0.63 | 1.00 | 36 | 13 |
| Plasma Protein-C | 0 | -0.56 | 0.56 | 1.00 | 17 | 42 |

**Table 2:** SMD and confidence intervals for biomarkers reported during hospitalisation in 1 study only, reported by timepoint

When one study reported; meta-analysis not possible therefore reported as SMD and confidence interval calculation. **Abbreviations:** SMD = standardised mean difference; LL = lower limit; UL = upper limit; CC16 = club cell protein 16; nCD64 = neutrophil cluster of differentiation 64; PAI-1 = plasma activator inhibitor 1; PCT= procalcitonin; PPC = plasma protein C; SPD = surfactant protein D; FGF = fibroblast growth factor-basic; IL = interleukin; TNF = tumor necrosis factor; TREM = triggering receptor expressed on myeloid cells; KL-6/MUC1 = Krebs von den Lungen 6/Mucin 1; BTLA = B and T lymphocyte attenuator; CD = cluster of differentiating; CTLA-4 = cytotoxic T lymphocyte antigen-4; ), GITR = glucocorticoid-induced tumor necrosis factor receptor-related protein; GITRL = GITR ligand; HVEM = herpes virus entry mediator; ICOS = inducible co-stimulatory molecule; LAG-3 = lymphocyte activation-gene-3; PD-1 = programmed cell death protein 1; PD-L1 = programmed cell death ligand-1; TIM-3 = t-cell immunoglobulin and mucin-domain containing-3; TLR-2 = toll-like receptor 2; sST2 = Soluble suppression of tumorigenicity-2; WBC = white blood cells; VAP = ventilator acquired pneumonia; NR = not recorded

| Biomarker | Time Point | Effect Size (SMD) | Lower Limit | Upper Limit | *p* | Sample size  Pneumonia | Sample size  No-Pneumonia |
| --- | --- | --- | --- | --- | --- | --- | --- |
| Angiopoietin |  |  |  |  |  |  |  |
|  | Day 2 | -0.02 | -0.54 | 0.49 | 0.93 | 20 | 53 |
|  | Day 5 | -0.08 | -0.59 | 0.44 | 0.77 | 20 | 53 |
| Antithrombin |  |  |  |  |  |  |  |
|  | Day 1-2 | -0.32 | -1.15 | 0.51 | 0.46 | 9 | 15 |
|  | Day 3-6 | -1.16 | -2.05 | -0.27 | 0.01 | 9 | 15 |
| CC16 |  |  |  |  |  |  |  |
|  | Day 2 | 0.86 | 0.41 | 1.30 | 0.001 | 30 | 71 |
|  | Pneumonia Onset | -1.14 | -1.72 | -0.56 | >0.001 | 20 | 38 |
| nCD64 |  |  |  |  |  |  |  |
|  | Pneumonia Diagnosis | -1.62 | -2.33 | -0.91 | >0.001 | 36 | 13 |
|  | Day 3 | 0 | -0.63 | 0.63 | 1 | 36 | 13 |
| PAI-1 |  |  |  |  |  |  |  |
|  | Day 1 | 1.56 | 0.80 | 2.32 | >0.001 | 12 | 28 |
|  | Day 3 | -0.17 | -0.85 | 0.51 | 0.62 | 12 | 28 |
|  | Day 5 | 1.12 | 0.40 | 1.84 | 0.002 | 12 | 28 |
|  | Day 7 | 1.79 | 1.01 | 2.57 | >0.001 | 12 | 28 |
|  | Day 10 | 2.41 | 1.56 | 3.27 | >0.001 | 12 | 28 |
|  | Day 14 | 0.37 | -0.31 | 1.05 | 0.29 | 12 | 28 |
|  | Day 21 | 0.89 | 0.19 | 1.59 | 0.01 | 12 | 28 |
| PCT |  |  |  |  |  |  |  |
|  | Pneumonia Diagnosis | 0.20 | -0.44 | 0.83 | 0.54 | 36 | 13 |
|  | Day 3 | -0.20 | -0.84 | 0.43 | 0.53 | 36 | 13 |
| Pentraxin |  |  |  |  |  |  |  |
|  | Day 2 | 0.07 | -0.45 | 0.58 | 0.80 | 20 | 53 |
|  | Day 5 | -0.17 | -0.68 | -0.35 | 0.53 | 20 | 53 |
| PPC |  |  |  |  |  |  |  |
|  | 6-hours | -2.04 | -2.72 | -1.37 | >0.001 | 17 | 42 |
|  | 12-hours | -2.74 | -3.49 | -1.99 | >0.001 | 17 | 42 |
|  | Day 1 | -3.16 | -3.96 | -2.36 | >0.001 | 17 | 42 |
| Blood Glucose |  |  |  |  |  |  |  |
|  | 8 days pre-VAP | 50.72 | 49.35 | 52.09 | >0.001 | 329 | 2327 |
|  | 6 days pre-VAP | -3.92 | -4.08 | -3.77 | >0.001 | 329 | 2327 |
|  | 2 days pre-VAP | -2.77 | -2.91 | -2.64 | >0.001 | 329 | 2327 |
|  | 3 days after-VAP | -1.02 | -1.14 | -0.90 | >0.001 | 329 | 2327 |
|  | 6 days after-VAP | -1.42 | -1.54 | -1.30 | >0.001 | 329 | 2327 |
|  | 8 days after-VAP | -1.73 | -1.85 | -1.60 | >0.001 | 329 | 2327 |
|  | 9 days after-VAP | -1.92 | -2.04 | -1.79 | >0.001 | 329 | 2327 |
|  | 10 days after-VAP | -2.36 | -2.49 | 2.23 | >0.001 | 329 | 2327 |
| Soluble Endothelial PPC |  |  |  |  |  |  |  |
|  | 6-hours | 0.89 | 0.30 | 1.47 | 0.003 | 17 | 42 |
|  | 12-hours | 0.86 | 0.27 | 1.44 | 0.004 | 17 | 42 |
|  | Day 1 | 0.16 | -0.40 | 0.73 | 0.57 | 17 | 42 |
| Soluble Thrombomodulin |  |  |  |  |  |  |  |
|  | 6-hours | 3.13 | 2.33 | 3.93 | >0.001 | 17 | 42 |
|  | 12-hours | 1.56 | 0.93 | 2.19 | >0.001 | 17 | 42 |
|  | Day 1 | 4.68 | 3.67 | 5.70 | >0.001 | 17 | 42 |
| SP-D |  |  |  |  |  |  |  |
|  | Day 1 | -0.74 | -1.61 | 0.13 | 0.10 | 8 | 16 |
|  | Day 2 | -0.02 | -0.45 | 0.40 | 0.91 | 30 | 71 |
| Creatinine | Day 1-2 | 0.18 | -0.87 | 1.23 | 0.74 | 7 | 7 |
| Bilirubin | Day 1-2 | -0.07 | -1.12 | 0.98 | 0.90 | 7 | 7 |
| FGF | NR | 0.21 | -0.49 | 0.91 | 0.55 | 9 | 64 |
| Gene Expression AHSP | VAP = Onset of VAP  No VAP = Day 5-10 | 0.03 | -0.32 | 0.38 | 0.87 | 41 | 124 |
| Gene Expression ALAS2 | VAP = Onset of VAP  No VAP = Day 5-10 | -0.23 | -0.59 | 0.12 | 0.20 | 41 | 124 |
| Gene Expression IL-1B | VAP = Onset of VAP  No VAP = Day 5-10 | -0.78 | -1.14 | -0.42 | >0.001 | 41 | 124 |
| Gene Expression IL-1RN | VAP = Onset of VAP  No VAP = Day 5-10 | -0.46 | -0.82 | -0.10 | 0.01 | 41 | 124 |
| Gene Expression IL-6R | VAP = Onset of VAP  No VAP = Day 5-10 | -1.18 | -1.55 | -0.80 | >0.001 | 41 | 124 |
| Gene Expression PCSK1 | VAP = Onset of VAP  No VAP = Day 5-10 | -0.52 | -0.88 | -0.16 | 0.004 | 41 | 124 |
| Gene Expression PPBP | VAP = Onset of VAP  No VAP = Day 5-10 | -0.77 | -1.13 | -0.40 | >0.001 | 41 | 124 |
| Gene Expression SPARC | VAP = Onset of VAP  No VAP = Day 5-10 | 0.45 | 0.10 | 0.81 | 0.01 | 41 | 124 |
| Gene Expression TNF | VAP = Onset of VAP  No VAP = Day 5-10 | -0.75 | -1.11 | -0.38 | >0.001 | 41 | 124 |
| Gene Expression TREM | VAP = Onset of VAP  No VAP = Day 5-10 | 0 | -0.35 | 0.35 | 0.99 | 41 | 124 |
| Glucose | Day 1-2 | -0.41 | -1.47 | 0.65 | 0.45 | 7 | 7 |
| Haemoglobin | Day 1-2 | -0.22 | -1.27 | 0.83 | 0.68 | 7 | 7 |
| IL-2R | NR | 1.48 | 0.75 | 2.22 | >0.001 | 9 | 64 |
| IL-33 | Day 1 | 1.18 | 0.27 | 2.09 | 0.01 | 8 | 16 |
| IL-8 | Day 1 | 1.17 | 0.26 | 2.08 | 0.01 | 8 | 16 |
| KL-6/MUC1 | Day 2 | 0.01 | -0.42 | 0.44 | 0.96 | 30 | 71 |
| Lactate | Day 1-2 | -0.60 | -1.67 | 0.47 | 0.27 | 7 | 7 |
| Platelets | Day 1-2 | 0.15 | -0.90 | 1.20 | 0.78 | 7 | 7 |
| RAGE | Day 1 | 0.90 | 0.02 | 1.79 | 0.05 | 8 | 16 |
| Slope BTLA | Day 1-2 | 1.22 | 0.08 | 2.36 | 0.04 | 7 | 7 |
| Slope CD27 | Day 1-2 | -1.26 | -2.41 | -0.11 | 0.03 | 7 | 7 |
| Slope CD28 | Day 1-2 | 1.54 | 0.35 | 2.74 | 0.01 | 7 | 7 |
| Slope CD40 | Day 1-2 | -0.15 | -1.20 | 0.90 | 0.78 | 7 | 7 |
| Slope CD80 | Day 1-2 | 1.03 | -0.08 | 2.15 | 0.07 | 7 | 7 |
| Slope CD86 | Day 1-2 | 1.13 | 0 | 2.26 | 0.05 | 7 | 7 |
| Slope CTLA-4 | Day 1-2 | 1.57 | 0.37 | 2.77 | 0.01 | 7 | 7 |
| Slope GITR | Day 1-2 | 1.17 | 0.04 | 2.30 | 0.04 | 7 | 7 |
| Slope GITRL | Day 1-2 | 1.08 | -0.04 | 2.20 | 0.06 | 7 | 7 |
| Slope HVEM | Day 1-2 | -0.50 | -1.56 | 0.56 | 0.36 | 7 | 7 |
| Slope ICOS | Day 1-2 | 1.10 | -0.02 | 2.23 | 0.05 | 7 | 7 |
| Slope LAG-3 | Day 1-2 | -0.76 | -1.84 | 0.33 | 0.17 | 7 | 7 |
| Slope PD-1 | Day 1-2 | 1.35 | 0.19 | 2.51 | 0.02 | 7 | 7 |
| Slope PD-L1 | Day 1-2 | 1.66 | 0.44 | 2.87 | 0.01 | 7 | 7 |
| Slope TIM-3 | Day 1-2 | -0.62 | -1.70 | 0.45 | 0.25 | 7 | 7 |
| Slope TLR-2 | Day 1-2 | 1.25 | 0.10 | 2.39 | 0.03 | 7 | 7 |
| Sodium | Day 1-2 | -0.39 | -1.45 | 0.67 | 0.47 | 7 | 7 |
| sST2 | Day 1 | 2.07 | 1.04 | 3.10 | >0.001 | 8 | 16 |
| WBC | Day 1-2 | 0.85 | -0.24 | 1.94 | 0.13 | 7 | 7 |
